# A Glycolysis-Related Gene Signature Correlates With the Characteristics of the Tumor Immune Microenvironment and Predicts Prognosis in Patients With Hepatocellular Carcinoma

**DOI:** 10.3389/fmolb.2022.834976

**Published:** 2022-04-25

**Authors:** Jun Yang, Yuening Zhang, Jin Duan, Xiaojie Huang, Haibin Yu, Zhongjie Hu

**Affiliations:** ^1^ Phase Ⅰ Clinical Trial Ward, Beijing Youan Hospital, Capital Medical University, Beijing, China; ^2^ Beijing Youan Hospital, Capital Medical University, Beijing, China

**Keywords:** immune-related glycolysis gene, hepatocellular carcinoma, prognosis, immune microenvironment, PD-L1

## Abstract

**Aim:** To develop a glycolysis-related gene signature that correlated with the characteristics of the tumor immune microenvironment and had good predictive power for overall survival (OS) in hepatocellular carcinoma (HCC).

**Methods:** Gene expression profiles, RNA sequencing data, clinical characteristics and survival information for 407 patients with HCC and 58 healthy controls were downloaded from the TCGA database. GSEA 4.1.0 software was used to evaluate the glycolysis-related pathways enriched in HCC compared to normal liver tissue. Univariate Cox, Least Absolute Shrinkage, Selection Operator, and two-step multivariate Cox analyses were used to construct a glycolysis-related gene signature for prognostic prediction. The glycolysis-related gene signature was combined with clinical characteristics to generate a nomogram. Tumor-infiltrating immune cell profiles and PD-L1 protein expression in HCC tissues were investigated.

**Results:** The gene expression profiles of HCC tissues were enriched in glycolysis-related pathways. A glycolysis-related gene signature was used to categorize patients as high-risk or low-risk, where high-risk patients had significantly worse OS. Receiver operating characteristic curves confirmed the predictive capability of the glycolysis-related gene signature for OS (AUC >0.80). There was a significant difference in M0 macrophage (*p* = 0.017), dendritic cell (*p* = 0.043), B cell (*p* = 0.0018), CD4 T cell (*p* = 0.003), Treg (*p* = 0.01) and mast cell (*p* = 0.02) content and PD-L1 protein expression (*p* = 0.019) between HCC tissues in patients in the high-risk and low-risk groups.

**Conclusion:** We established a glycolysis-related gene signature for OS in HCC that was predictive in training and test TCGA cohorts and correlated with the characteristics of the HCC tumor immune microenvironment. The glycolysis-related gene signature may guide clinical decision-making concerning patient selection for immunotherapy in HCC.

## Introduction

Hepatocellular carcinoma (HCC) is the most prevalent form of liver cancer. HCC is susceptible to metastasis and a frequent cause of cancer-related mortality (Siegel et al., 2020). Treatments for HCC include partial liver resection, interventional surgery, liver transplantation, and systemic treatment (Hua et al., 2018). These have benefit in early-stage patients, but the treatment of patients with advanced HCC is challenging (Forner et al., 2018).

Cancer progression is promoted by aerobic glycolysis, which is persistently activated in tumor cells (Ganapathy-Kanniappan, 2018). The liver is essential for regulating glucose metabolism (Kitamura et al., 2011), and HCC cells are characterized by increased glycolysis and lactic acid production (Liberti and Locasale, 2016). There are several reports describing a glycolysis-related gene-signature that can predict prognosis in patients with HCC (Zhou et al., 2020).

Novel immuno-oncology drugs are changing the outcomes of patients with HCC (Cheng et al., 2019; Anwanwan et al., 2020). Immune cells act as tumor suppressors or promoters and play a pivotal role in the tumor microenvironment (Lei et al., 2020). The characteristics of the tumor microenvironment greatly impact the response to immunotherapy (Zhang et al., 2019). There remains a critical unmet need to identify immune-related indicators that can predict response to immunotherapy and prognosis in patients with HCC.

To fill this evidence gap, we studied the correlation of clinical characteristics with glycolysis-related gene expression, explored multiomics differences in clinical characteristics and immune infiltration status, and constructed a prognostic signature related to glycolysis and immunity that has effective predictive value in patients with HCC.

## Materials and Methods

### Data Source

Gene expression profiles, RNA sequencing data, clinical characteristics and survival information for 407 patients with HCC and 58 healthy controls were extracted from The Cancer Genome Atlas (TCGA) database. Patients were randomly divided into training and test cohorts. Gene expression profiles were merged and normalized using the “sva” package (Leek et al., 2019) in R (v4.1.0).

### Gene Set Enrichment Analysis of Glycolysis-Related Pathways

The Gene Set Enrichment Analysis **(**GSEA) website provided data on five glycolysis-related pathways. Glycolysis-related pathways enriched in HCC compared to normal liver tissue were identified using GSEA 4.1.0 software.

### Establishment of a Prognostic Glycolysis-Related Gene Signature

The performance of glycolysis-related genes for predicting survival in patients with HCC was assessed with Least Absolute Shrinkage, Selection Operator (LASSO) and multivariate Cox regression analyses using the “glmnet” package in R.

A prognostic glycolysis-related gene signature was established and a signature-based risk score was calculated for each patient, as: score = e^sum^ (mRNA level X multivariate Cox regression coefficient ratio of each mRNA). Using the median risk score as the cutoff value, patients were classified as high-risk or low-risk. Overall survival (OS) and gene expression profiles for high-risk and low-risk patients were determined using the “survival” and “pheatmap” packages in R. Kaplan–Meier survival and receiver operating characteristic (ROC) curve analyses were used to estimate the sensitivity and specificity of the glycolysis-related gene signature.

### Establishment of a Nomogram

A nomogram with independent predictors of prognosis was constructed. Goodness-of-fit of the nomgram was assessed with the area under the receiver operating characteristic curve and the Hosmer-Lemeshow test.

### Validation of the Prognostic Glycolysis-Related Gene Signature

The cutoff value for high-risk vs. low-risk patients and differences in OS and clinicopathological factors, such as age, sex and tumor-node-metastasis (TNM) stage, between the high-risk and low-risk groups were validated using Kaplan–Meier survival and ROC curve analyses. Associations between the glycolysis-related gene signature and clinicopathological parameters were assessed using the χ^2^ or Wilcoxon signed-rank test. The potential for the glycolysis-related gene signature to represent an independent predictor of prognosis was explored using univariate and multivariate Cox regression analyses. These operations used the survival, glmnet, pbapply, survival ROC, survminer, pHeatmap, and ggupbr packages in R.

### Tumor-Infiltrating Immune Cell Estimation

The association of the glycolysis-related genes with the HCC tumor microenvironment was explored by constructing a violin plot using the Fluidigm Singular Analysis Toolset 3.5.2 R package. This showed the contribution of immune cell infiltration in HCC tissues in patients in the high-risk and low-risk groups. Tumor-infiltrating immune cell (TIIC) profiles were assessed with scatter plots and Spearman correlations using the Tumor-Immune Estimation Resource (Li et al., 2017). TIICs in HCC tissues in patients in the high-risk and low-risk groups were compared using the limma and ggpubr packages in R.

### PD-L1 Protein Expression in the HPA Database

PD-L1 protein expression was investigated in healthy liver tissues and liver cancer tissues from the HPA dataset (http://www.proteinatlas.org/) by immune histochemical staining.

## Results

### Study Design and Summary of Patients’ Information

**FIGURE 1:**
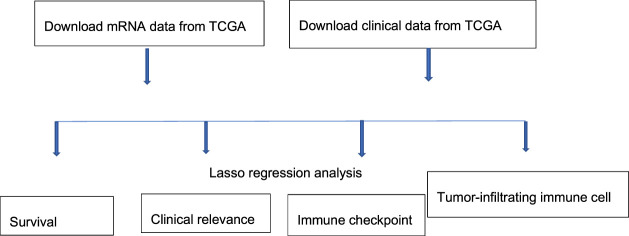
Study process.

**FIGURE 2:**
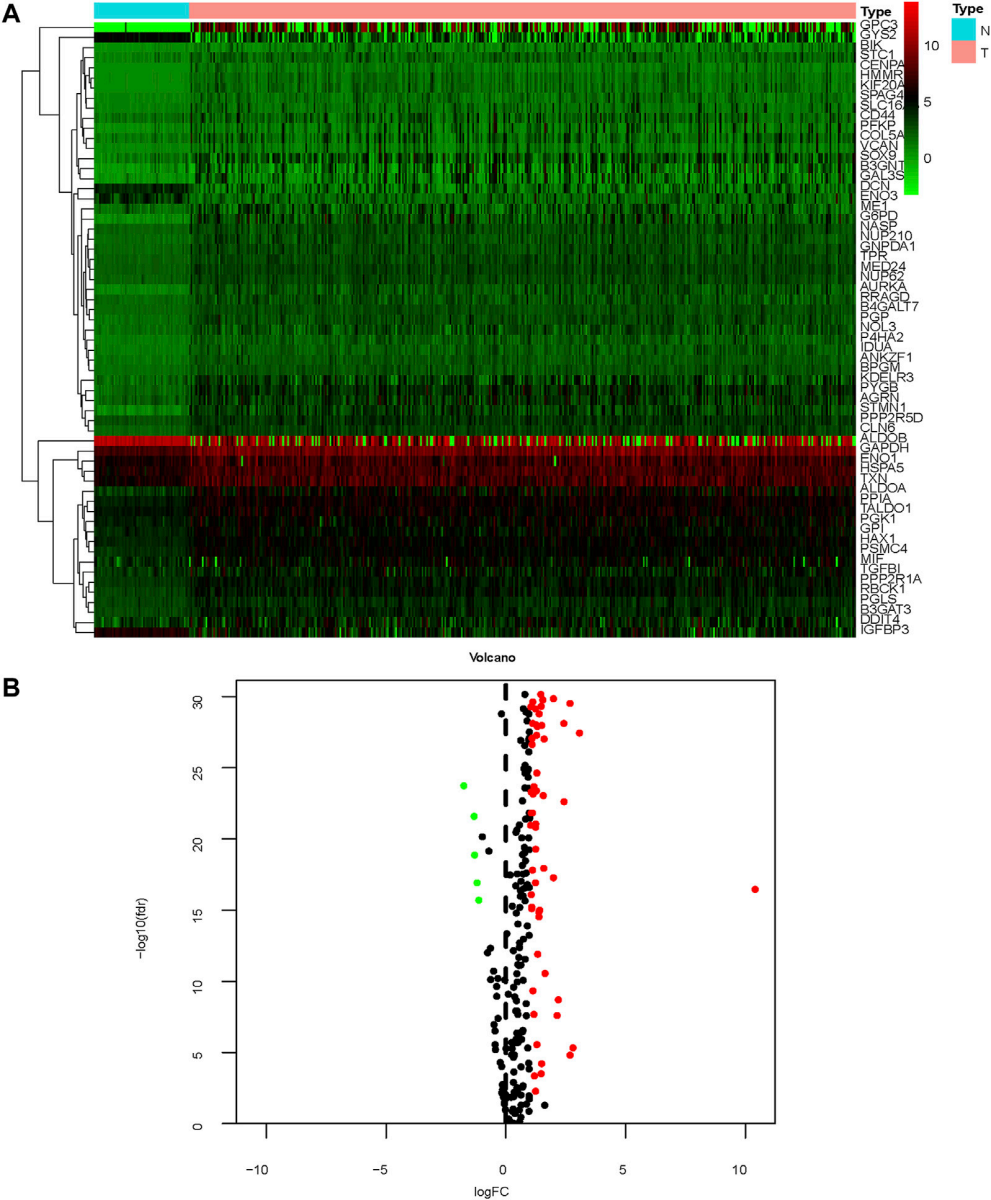
Gene expression profiles of patients with HCC **(A)** Heatmap; **(B)** mRNA volcano map.

The study process is shown in Figure 1. A total of 465 (*n* = 407 HCC tissues; *n* = 58 healthy liver tissues) gene expression profiles were obtained from the TCGA database (Table 1). An mRNA volcano map and an mRNA heatmap are shown in Figure 2.

**TABLE 1:** Baseline data of all HCC samples.

Characteristic	Type	Nunber	Proportion (%)
Age	≤65	235	57.74
	>65	141	34.64
	Unknown	31	7.6
Gender	Female	146	35.87
	Male	261	64.13
Grade	G1-2	235	57.73
	G3-4	137	33.66
	Unknown	35	8.6
Stage	Stage I–II	292	71.74
	Stage III–IV	102	25.06
	Unknown	13	3.2
T Stage	T1–2	311	77.15
	T3–4	94	23.1
	Unknown	2	0.5
M Stage	M0	303	74.45
	M1	8	1.97
	Unknown	96	23.59
N stage	N0	290	71.25
	N1	8	1.97
	Unknown	109	26.78

### GSEA of Glycolysis-Related Pathways

**FIGURE 3:**
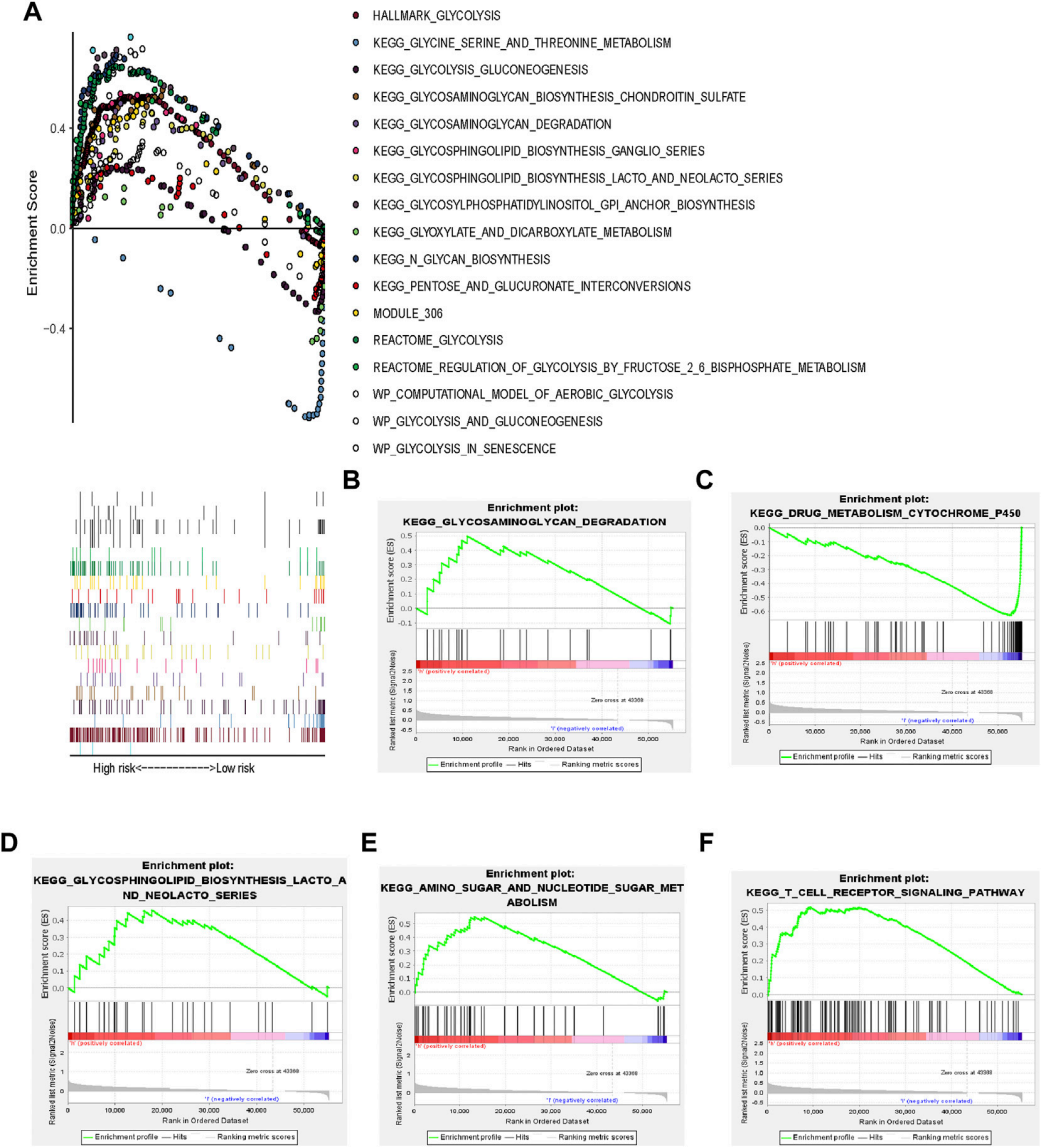
Glycolysis-related gene signature **(A)** GSEA of 17 pathways; **(B–F)** KEGG pathways.

HCC tissues were enriched in glycolysis-related genes (Figure 3). The top 17 Kyoto Encyclopedia of Genes and Genomes pathways are shown in Figure 3A.

### Construction of a Prognostic Glycolysis-Related Gene Signature

**FIGURE 4:**
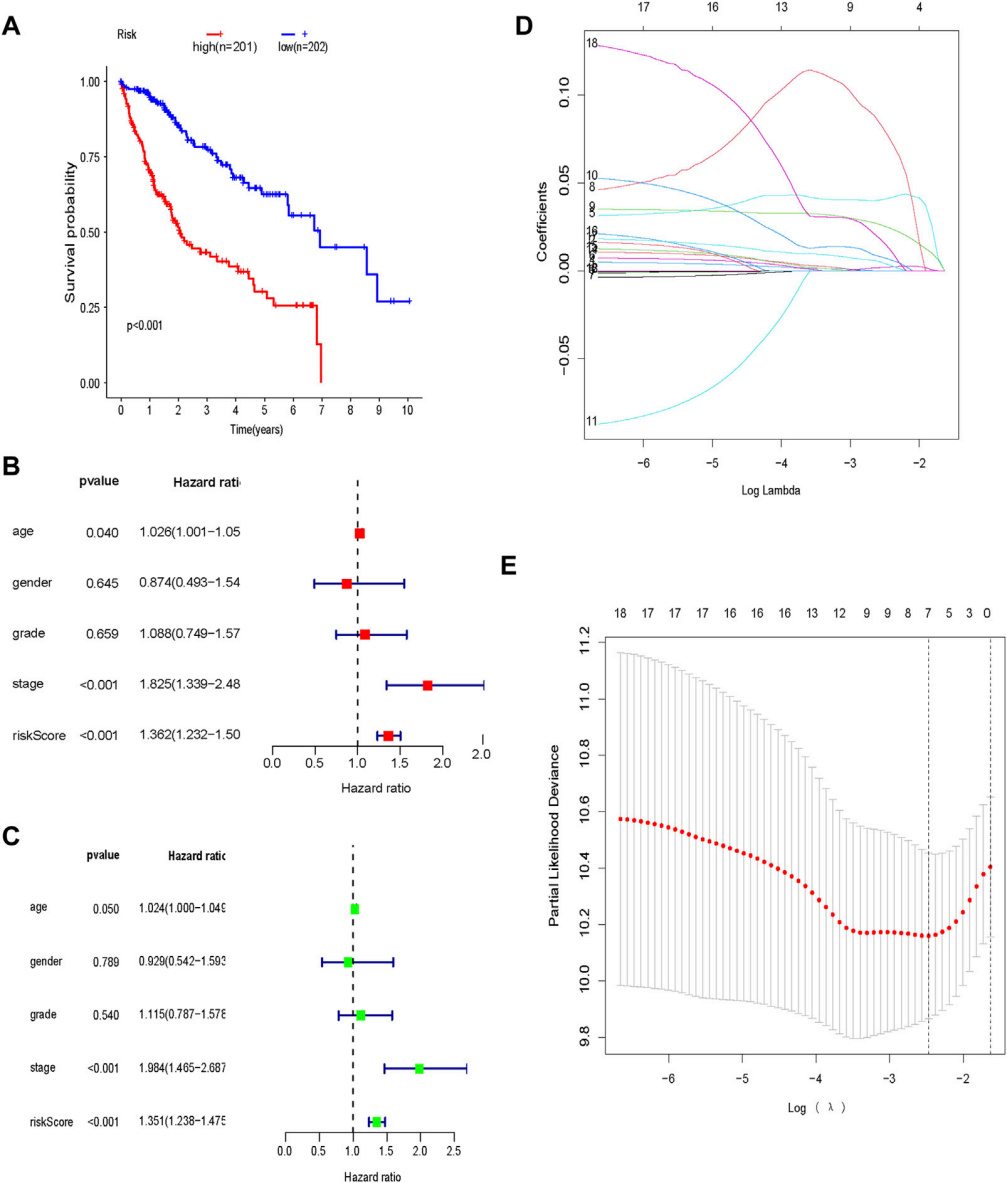
Prognostic glycolysis-related gene signature **(A)** Kaplan–Meier survival analysis of the low-risk and high-risk groups; **(B)** Univariate Cox regression analysis of the risk score and clinical characteristics; **(C)** Multivariate Cox regression analysis of the risk score and clinical characteristics; **(D)** LASSO regression; **(E)** Optimal lambda values.

The expression profiles of the glycolysis-related genes were incorporated into a prognostic indicator using LASSO Cox regression analysis (Figures 4D,E). A glycolysis-related gene signature was constructed. All patients in the TCGA dataset were classified as high-risk (*n* = 202) or low-risk (*n* = 201) based on their signature-based risk scores. OS was significantly longer in low-risk patients compared to high-risk patients (Figure 4A). Univariate and multivariate analyses indicated that the glycolysis-related gene signature was an independent predictor of OS and several clinicopathological factors, including age, sex and tumor stage, in HCC (Figures 4B,C).

### Validation of the Prognostic Glycolysis-Related Gene Signature

**FIGURE 5:**
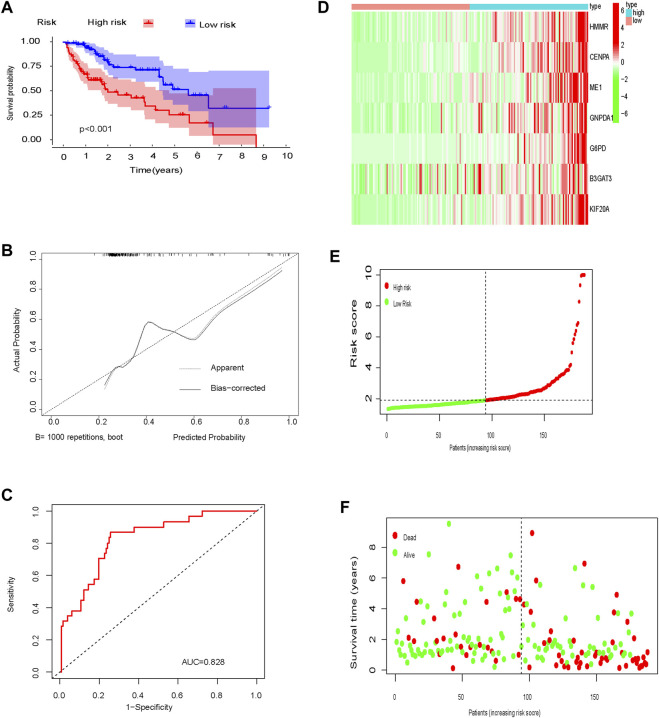
Training set **(A)** Kaplan-Meier curve; **(B)** Calibration curve; **(C)** Time-dependent ROC curve analysis; **(D)** Heat map of glycolysis-related gene expression; **(E)** Distribution of risk scores; **(F)** Survival time.

In the training cohort, OS was significantly longer in low-risk patients compared to high-risk patients (*p* < 0.001; Figure 5A). Calibration curves showed good consistency between the prediction based on the glycolysis-related gene signature and the actual observation (Figure 5B). The predictive ability of the glycolysis-related gene signature, determined by the area under the ROC curve (AUC = 0.828; Figure 5C), was good. The distribution of risk scores, patient survival status, and a heatmap of glycolysis-related gene expression are shown in Figure 5F.

**FIGURE 6:**
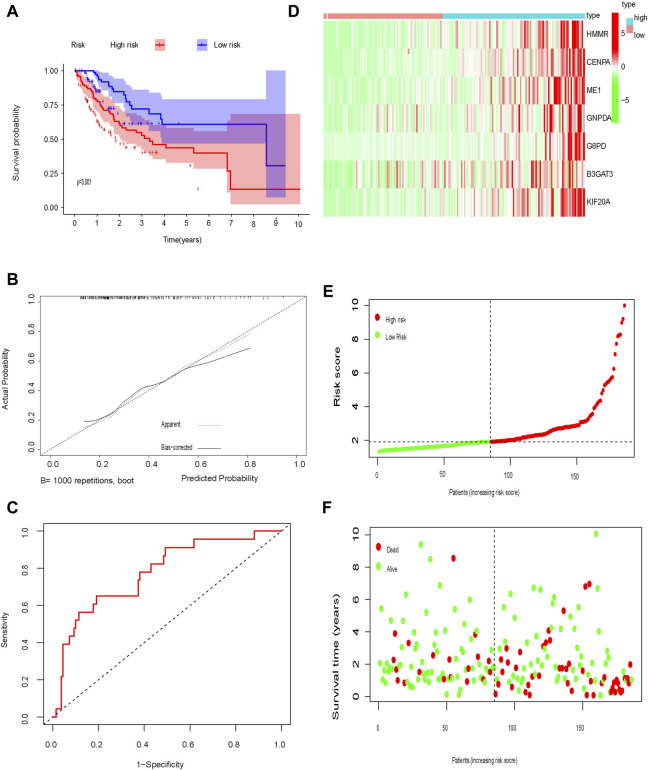
Test set **(A)** Kaplan-Meier curve; **(B)** Calibration curve; **(C)** Time-dependent ROC curve analysis; **(D)** Heat map of glycolysis-related gene expression; **(E)** Distribution of risk scores; **(F)** Survival time.

The test cohort was used to validate the prognostic glycolysis-related gene signature. OS was significantly longer in low-risk patients compared to high-risk patients (*p* = 0.001; Figure 6A). ROC curves, the distribution of risk scores, patient survival status, and a heatmap of glycolysis-related gene expression are shown in Figures 6B–F.

### Stratification of the Prognostic Glycolysis-Related Gene Signature

**FIGURE 7:**
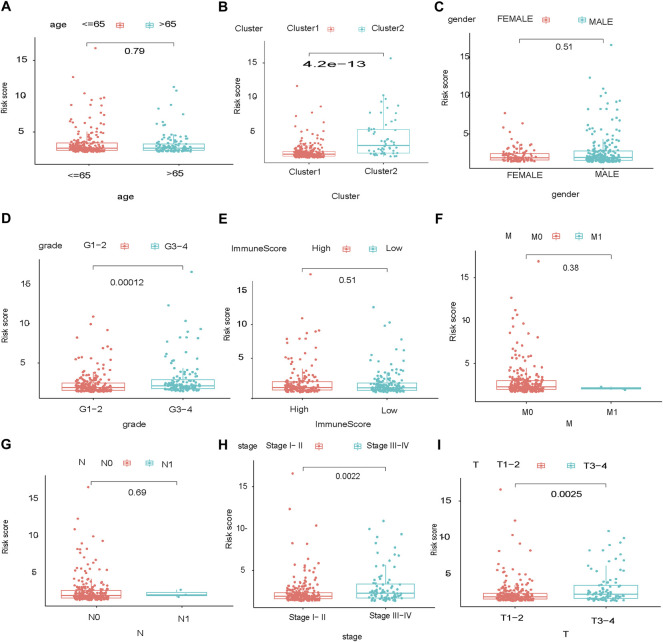
Stratification of the prognostic glycolysis-related gene signature. Stage of HCC: TMN, high:high risk, low:low risk.

The glycolysis-related gene signature was significantly associated with tumor stage (*p* = 0.0022), the T category of TNM staging (*p* = 0.0025) and tumor grade (*p* = 0.00012), but not the M category of TNM staging (*p* = 0.38), N category of TNM staging (*p* = 0.69), sex (*p* = 0.51), or age (*p* = 0.79) (Figures 7A–I).

### Nomogram Building and Validation

**FIGURE 8:**
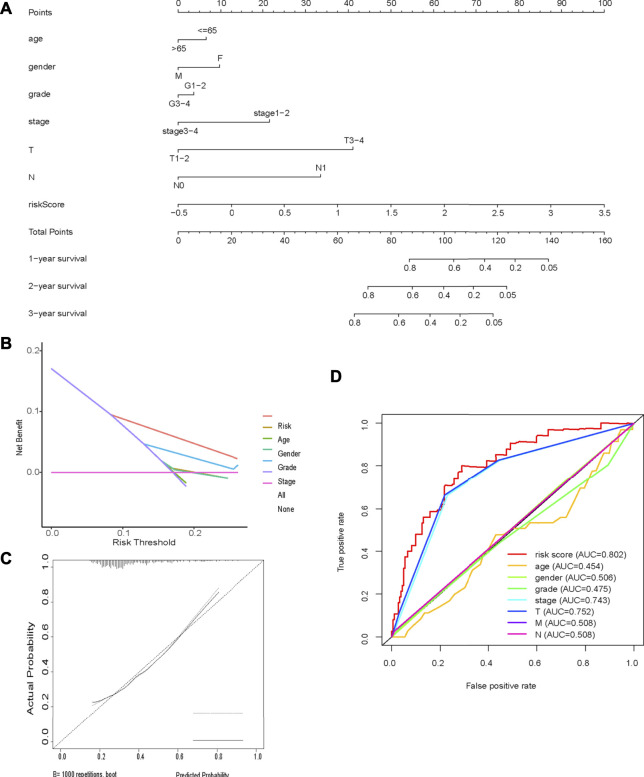
Nomogram **(A)** Nomogram incorporating prognostic factors **(B)** Decision curve analysis; **(C)** Calibration curves showed good consistency between the nomogram prediction and the actual observation; **(D)** Time-dependent ROC curve analysis.

Multivariate Cox regression analysis identified the risk score, age, sex, and tumor stage as independent prognostic factors in the TCGA cohort. A novel nomogram incorporating these prognostic factors was constructed. The risk score had the widest range of variables when calculating the total nomogram score, indicating that it played a prominent role in determining OS (Figure 8A). Calibration curves showed good consistency between the nomogram prediction and the actual observation (Figure 8C). The predictive ability of the nomogram, determined by the area under the ROC curve (AUC = 0.80; Figure 8D), was good. Decision curve analysis indicated that the nomogram had the best net benefit (Figure 8B).

### Tumor Infiltration Immune Cell Profiling

**FIGURE 9:**
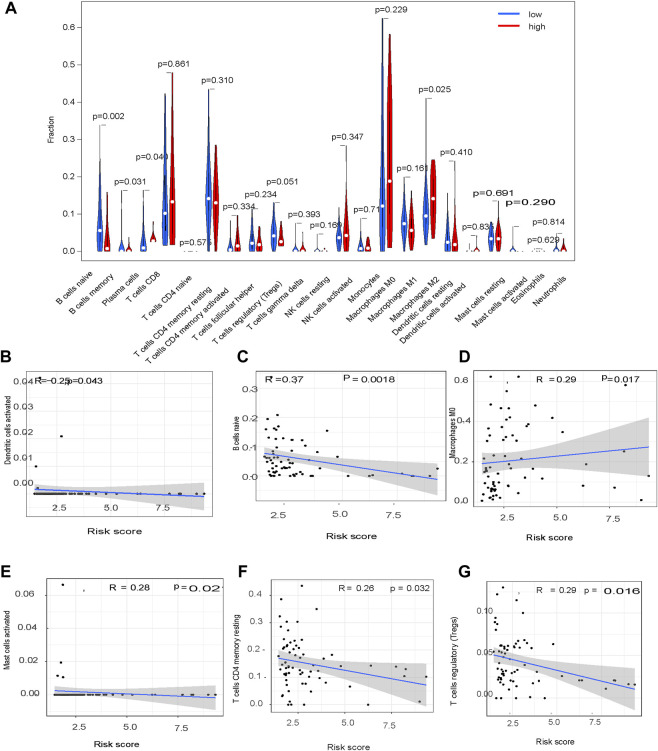
Tumor infiltration immune cell profiling.

CIBERSORT showed a significant difference in M0 macrophage (*p* = 0.017), dendritic cell (*p* = 0.043), B cell (*p* = 0.0018), CD4 T cell (*p* = 0.003), Treg (*p* = 0.01) and mast cell (*p* = 0.02) content in HCC tissues from patients in the high-risk group compared to the low-risk group (Figures 9A–G).

### Verification of PD-L1 Protein Expression in the HPA Database

**FIGURE 10:**
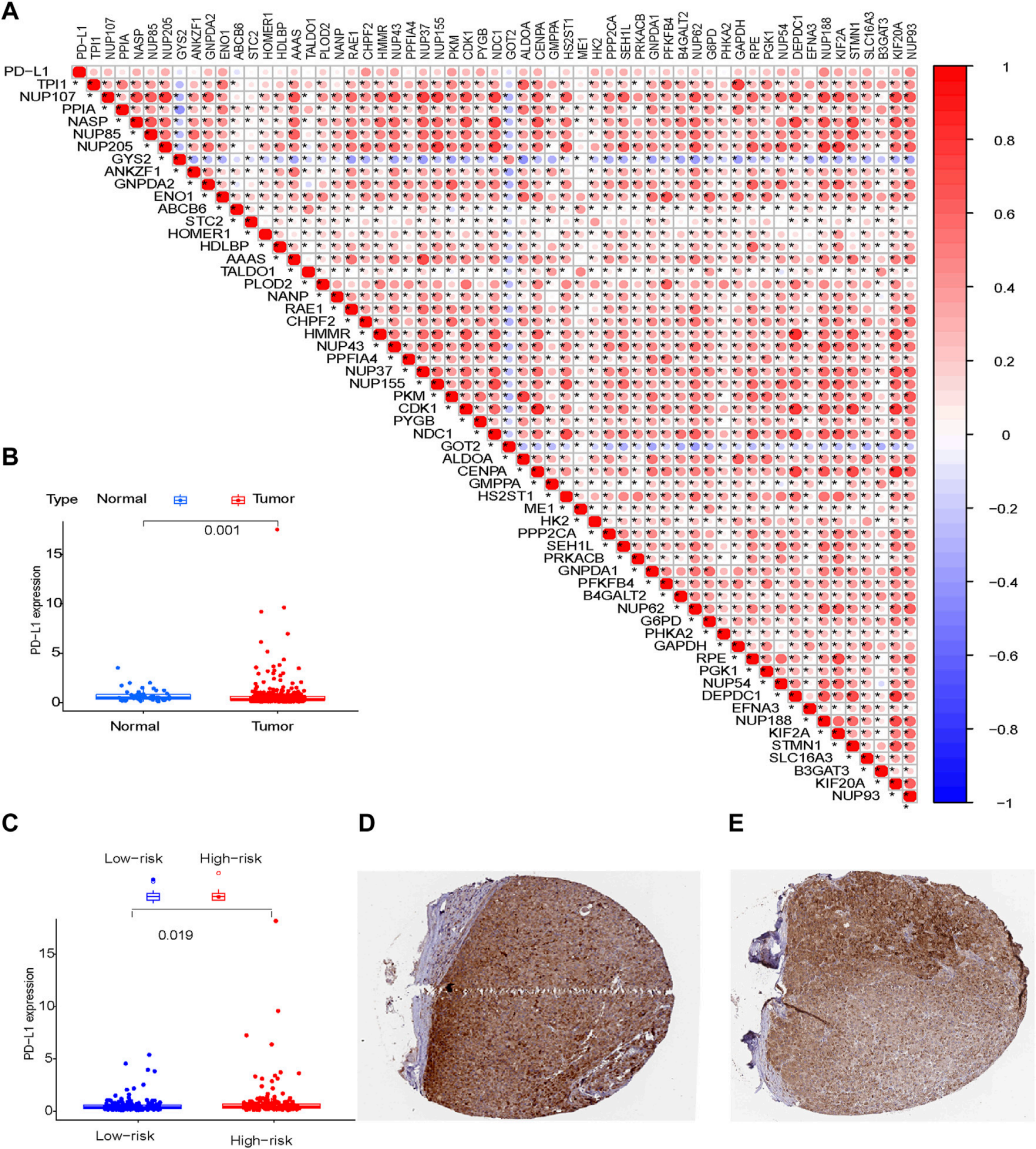
PD-L1 protein expression **(A)** Risk model; **(B)** Normal tissue and tumor tissue; **(C)** Tumor tissues from patients in the low-risk and high-risk groups; **(D,E)** Tumor tissues derived from the HPA database.

Immunohistochemical staining showed a significant difference in PD-L1 expression level between normal liver tissue and liver cancer tissue (Figure 10B; *p* = 0.001) from the HPA dataset (http://www.proteinatlas.org/) and HCC tissues from patients in the high-risk group compared to the low-risk group in the TCGA cohort (Figure 10C **;** *p* = 0.019).

## Discussion

HCC has a complex pathogenesis and involves dysregulation of the cell cycle and energy metabolism (Hsu et al., 2015). mRNAs are involved in tumorigenesis and progression by regulating the rate-limiting enzymes involved in energy metabolism. Glycolysis is an inefficient form of energy generation, but it produces reducing equivalents (Terabe et al., 2019) and biosynthetic substrates required for tumor cell proliferation (Liang et al., 2019). The objective of this study was to develop a novel prognostic glycolysis-related gene signature that correlated with the characteristics of the tumor immune microenvironment in HCC. Gene expression profiles and clinical information were obtained from the TCGA database. Glycolysis-related genes were identified using univariate Cox, LASSO, and two-step multivariate Cox analyses. Differentially expressed glycolysis-related genes were combined to construct a glycolysis-related gene signature with value for predicting prognosis. The glycolysis-related gene-signature was combined with clinical characteristics to generate a nomogram with a high prediction performance. The glycolysis-related gene signature correlated with TIIC profiles and PD-L1 protein expression in HCC tissues. Previous reports have described glycolysis-related gene-signatures that can predict prognosis in patients with HCC; however, our glycolysis-related gene-signature has greater capability for predicting survival (Zhou et al., 2020).

Glycolysis-related genes may represent biomarkers of survival and prognosis in HCC, likely because altered energy metabolism and epigenetic processes are key components of HCC pathophysiology (Chen et al., 2018). Specifically, the Warburg effect, characterized by high glucose uptake and increased lactate production, is a phenotype commonly exhibited by HCC cells (Anderson et al., 2018; Pascale et al., 2020). The Warburg effect results from mitochondrial dysfunction (Riera Leal et al., 2020), tumor adaptation (Ždralević et al., 2018), microenvironmental changes (Sun et al., 2018), oncogenes (Banks, 2013) and abnormalities in related signaling pathways. In the present study, GSEA showed HCC tissues from patients at high risk for poor prognosis were enriched in glycolysis-related genes. Glycolysis-related pathways have been associated with recurrence in liver cancer (Zhang et al., 2018).

A previous study reported that long coding RNAs were involved in HCC immune evasion (Peng et al., 2020). Consistent with this, findings from the present study showed a significant difference in the immune cell profile of HCC tumors in the high-risk and low-risk groups, risk characteristics could be used as immune indicators for HCC, immune features were activated when the risk score increased, and the proportion of Tregs increased with an increase in the risk score. These data indicate Tregs play a role in the HCC tumor microenvironment, and patients with low-risk scores may respond to immunotherapy.

TIICs have been identified as a key component of the tumor microenvironment and response to immunotherapy. An immune score is usually used to guide immunotherapy and assess prognosis. Immune checkpoint inhibitors have changed the therapeutic landscape in cancer (Salik et al., 2020). PD-L1 blockade has provided new insights into the clinical management of HCC (Ng et al., 2020). mRNAs play a key role in immune regulation, through antigen presentation and immune cell infiltration (Denaro et al., 2019). In the present study, there was a significant difference in PD-L1 expression level in HCC tissues from patients in the high-risk group compared to the low-risk group, suggesting patients with high-risk scores may be candidates for immunotherapy targeting the PD-1/PD-L1 pathway.

This study was limited. First, it was a retrospective analysis using information extracted from a single database. we are going to examine the conclusion with our own patient cohort in future. Second, in Figure 7B, although *p* value <0.05, the Pearson R value are mostly lower than 0.3. According to the data, it is hard to drawn any conclusion about the relationship between glycolysis-related pathway and immune infiltration. We are going to study the question in other data in future. Third, we are going to examine the impacts of genes involved in glycolysis-related signature on tumor progression and immunotherapy efficacy by establishing specific gene knockout hepatoma-bearing mice in future.

Future research should be large scale, prospective, and involve multiple centers.

## Conclusion

We established a glycolysis-related gene signature for OS in HCC that correlated with the characteristics of the HCC tumor immune microenvironment and was predictive in training and test TCGA cohorts. The glycolysis-related gene signature may guide clinical decision-making concerning patient selection for immunotherapy in HCC.

## Data Availability

The datasets presented in this study can be found in online repositories. The names of the repository/repositories and accession number(s) can be found in the article/Supplementary Material.
